# Changing patterns and clinical outcomes of hospitalized patients with COVID-19 severe pneumonia treated with remdesivir according to vaccination status: results from a real-world retrospective study

**DOI:** 10.1007/s10238-023-01036-x

**Published:** 2023-03-24

**Authors:** Daniele Mengato, Maria Mazzitelli, Andrea Francavilla, Monica Bettio, Lolita Sasset, Nicolò Presa, Lisa Pivato, Sara Lo Menzo, Marco Trevenzoli, Francesca Venturini, Dario Gregori, Anna Maria Cattelan

**Affiliations:** 1 University of Padua, Padua, Italy; 2 Padova University Hospital, Hospital Pharmacy Unit, Padua, Italy; 3 Padova University Hospital, Infectious and Tropical Diseases Unit, Padua, Italy; 4 Department of Cardiac Thoracic Vascular Sciences and Public Health, University of Padua, Padua, Italy; 5 Department of Molecular Medicine, University of Padua, Padua, Italy

**Keywords:** COVID-19, Remdesivir, SARS-CoV-2, Mortality, Real-world data

## Abstract

Since the beginning of Coronavirus Disease 2019 (COVID-19) pandemic, many drugs have been purposed for the treatment of severe acute respiratory syndrome-coronavirus-2 (SARS-CoV-2). Remdesivir emerged as an encouraging antiviral drug for patients with documented severe COVID-19-related pneumonia. Although several studies about remdesivir effectiveness exist, no study investigated the effect of the combination of remdesivir with the vaccination status. The aim of this study was to assess whether the administration of remdesivir could show some differences in terms of clinical outcomes in patients vaccinated against SARS-CoV-2 versus those who were not. The primary outcome was the in-hospital mortality. The secondary outcomes were 30-days mortality, the need for ICU admission and for oxygen supplementation. This is a retrospective cohort study including all consecutive adult patients hospitalized for severe COVID-19 at the Padua University Hospital (Italy), between September 1st, 2020, and January 31st, 2022, and who received a 5-days course of remdesivir. A total of 708 patients were included, 467 (66%) were male, and the median age was 67 (IQR: 56-79) years. To better estimate the outcomes of interest, a propensity score weighted approach was implemented for vaccination status. A total of 605/708 patients (85.4%) did not complete the vaccination schedule. In-hospital mortality rate was 5.1% (*n* = 36), with no statistically significant difference between the unvaccinated (n=29, 4.8%) and vaccinated (n=7, 6.8%; p=0.4) patients. After propensity score matching, mortality between the two groups remained similar. However, both the need for ICU and oxygen supplementation were significantly lower in the vaccinated group. Our finding suggests that a complete vaccination course could have an impact in reducing the need for transfer in ICU and for high-flow therapy in moderate-to-severe COVID-19 patients treated with remdesivir.

## Introduction

Coronavirus disease 2019 (COVID-19), caused by the severe acute respiratory syndrome coronavirus-2 (SARS-CoV-2), affected, as of September 2022, 600 million people worldwide, causing a devastating pandemic with more than 6 million deaths [1, 2].

Since the beginning of the pandemic, two main types of approaches have been implemented: a primary prevention strategy (i.e., vaccination) and the searching of an effective treatment [3].

About the former, in late December 2020, the first vaccine became available [4]. Since then, a vaccination program gradually became the main answer for primary prevention purposes [5]. With regard to the treatment of patients with severe COVID-19 pneumonia, multiple and different drugs have been used [6–8]. However, a lot of questions were raised and often clinical trials drawn conflicting results. As a consequence, several treatments have been proposed over time, and the reversal of a previously recommended drug was not infrequent [9].

In this context, remdesivir, an adenosine analogue with a broad-spectrum antiviral activity against RNA viruses, was one of the most promising drugs for the treatment of COVID-19-related pneumonia [10]. Data from randomized clinical trials (RCTs) demonstrated that remdesivir was associated with a significantly faster recovery time and with a lower mortality rate [11]. Therefore, it received the US Food and Drug Administration (FDA) full approval in October 2020 for COVID-19 treatment and a moderate recommendation by the National Health Institute (NHI) COVID-19 guidelines [12]. In Italy due to the severity of the second pandemic wave, it quickly became a widely used therapeutic option, accounting for an overall amount of 80.000 treatments until February 2022 [13].

Several factors, such as the incidence of new variants of concern (VOCs), the availability of vaccines, the emerging of new therapies for early disease stages (e.g., monoclonal antibodies), and advanced disease stages (e.g., tocilizumab, baricitinib, and anakinra), may have influenced the outcomes of patients hospitalized with severe COVID-19 pneumonia [15–18].

However, the findings from the Solidarity trial and from a recently updated meta-analysis did not provide clear evidence of the efficacy of remdesivir on COVID-19 mortality, need for mechanical ventilation, time to clinical recovery, and other patient-related outcomes [19, 20]. This new evidence led the World Health Organization (WHO) to a conditional recommendation against the use of remdesivir in hospitalized patients [21]. The focus on different outcomes, the analysis of subgroups of patients, and the potential influence of the timing of remdesivir after the onset of symptoms could partially contribute to these controversial results [22]. It is also interesting to study the role of remdesivir in a SARS-CoV-2 vaccinated population. In fact, to date, no specific data are available on the possible correlation between vaccination status and antiviral therapy.

Since December 2020, a massive vaccination campaign has begun worldwide, and to date, the epidemiological data has shown the excellent efficacy of the different vaccine methods, both mRNA- and viral vector-based, in the prevention from symptomatic SARS-CoV-2 infections, SARS-CoV-2 hospitalization, severe disease, and death.

Even though, thanks to vaccination, number of hospitalizations and cases of severe COVID-19 constantly declined, real-world data on the potential synergistic interaction between remdesivir and SARS-CoV-2 vaccination are still lacking.

Therefore, given the ongoing pandemic, a retrospective observational study was conducted at the Department of Infectious Diseases of the Padua University Hospital, Italy, to describe the real-world effectiveness of a 5-day course of remdesivir for the treatment of severe pneumonia according to SARS-CoV-2 vaccination status.

## Methods

This retrospective cohort study included all consecutive adult patients hospitalized for moderate or severe COVID-19 at Infectious and Tropical Diseases Department (located in a third level center, with 1.600 beds), between September 1st, 2020, and January 31st, 2022, and who received remdesivir at a scheduled dosage of 200 mg the 1st, followed by and 100 mg/day for the following 4 days. All patients required supplemental oxygen with nasal cannula, and all had radiographic evidence of interstitial pneumonia. In case of rapid clinical deterioration, the decision process of upgrade ventilation support was guaranteed by a multidisciplinary team (infectious diseases physician, pneumologists, and critical care physician) specifically implemented in our hospital during the COVID-19 pandemic.

As per the Italian Medicines Agency (AIFA) recommendations, patients presenting at admission need for high-flow nasal cannula, non-invasive or invasive mechanical invasive ventilation, vasoactive drugs, extracorporeal membrane oxygenation (ECMO), or with multiorgan failure were excluded, as well as patients with one or more remdesivir-related contraindications. The latters included elevations of liver function test > 5 times the upper normal limit, estimated glomerular filtration rate below 30 ml/minute, need for haemodialysis or peritoneal dialysis, and being pregnant or breastfeeding.

We retrieved the following data from medical electronic health records (EHR): demographics (age, sex, and nationality), vital signs, comorbidities, comedications, vaccination status against SARS-CoV-2, laboratory data, remdesivir treatment duration, type of oxygen support, and possible worsening status (e.g., HFNC, non-invasive positive pressure ventilation [NIPPV], IMV, ECMO, and admission to intensive care unit [ICU]), length of hospital stay, type of discharge, and outcome. These data were cross-checked with data from an integrated analytics application (Qlikview®, QlikTech International AB, King of Prussia, USA) and data from AIFA's Monitoring Registers [23]. All data were collected on an electronic spreadsheet.

### Outcomes

Clinical outcomes were categorised as death, discharge at home, and transfer to another health-care facility. The primary outcome of this study was the mortality rate of patients during hospitalization stratified by vaccination status. The secondary outcomes were the mortality within 30 days, the rate of ICU’s admissions, the need for high-flow oxygen (HFO) supplementation, and the incidence of serious adverse reactions documented by the local pharmacovigilance authority. The secondary outcomes were analyzed according to the vaccination status as well.

### Statistical analysis

Continuous data were reported as median and interquartile range (IQR) and compared using the Mann—Whitney test. Categorical variables were reported as frequencies and percentages and analyzed with the χ^2^-test with Yates' correction or Fisher’s exact test, as appropriate. A propensity score matching was implemented to estimate the role of full vaccination in this cohort of patients, with the attempt of minimizing the patients’ selection bias. For the propensity score estimation, a ratio of 1:1 was adopted, and a logit function was specified to derive the associated probabilities. When used in cohort studies, the propensity score represents a valid statistical option, since it allows patients with similar characteristics to be compared even in the absence of direct comparisons previously identified in the study design.

All data were analyzed through the R System [24]. The following variables were used to estimate propensity score: age, gender, comorbidities, days between the onset of symptoms and administration of remdesivir and monoclonal antibodies (MABs).

## Results

Over the study period, 708 patients with COVID-19 pneumonia and who received remdesivir were included. Patient’s characteristics are depicted in Table 1. The median age was 67 (IQR: 56-79) years; 467 (66.0%) were male, and 86 (12.1%) were from other nationalities than Italian. Five hundred and fifty-six patients (78.5%) had at least one comorbidity, and 222 (31.4%) patients had three or more concomitant diseases. The most prevalent comorbidity was hypertension (*n* = 358, 50.6%), followed by other cardiovascular diseases (*n* = 275, 38.8%) and diabetes (*n* = 142, 20.1%).

**Table 1:** Patients’ characteristics at baseline

	*N* = 708
Age, median (IQR)	67 (56, 79)
Gender	
Female, n (%)	241 (34%)
Male, n (%)	467 (66%)
Nationality	
Italia, n (%)	622 (88%)
Foreign country, n (%)	86 (12%)
Days from symptoms to remdesivir, median (IQR)	7.0 (4.0, 9.0)
*Days from symptoms to remdesivir*	
< = 5, n (%)	256 (36%)
> 7, n (%)	231 (33%)
6–7, n (%)	221 (31%)
*Variant of concern (VOC)*	
Native, n (%)	378 (54%)
Alpha (B.1.1.7), n (%)	155 (22%)
Delta (B.1.617.2), n (%)	116 (16%)
Omicron “family” (BA.1, BA.2, BA.4, and BA.5), n (%)	59(8%)
Comorbidities, at least 1, n (%)	556 (79%)
Comorbidities, three or more, n (%)	221 (31%)
Hypertension, n (%)	357 (50%)
Diabetes, n (%)	142 (20%)
Obesity, n (%)	84 (12%)
Neoplasms, n (%)	102 (14%)
Cardiac diseases, n (%)	275 (39%)
Chronic respiratory diseases, n (%)	57 (8.0%)
Other comborbidities, n (%)	245 (35%)
*COVID-19 vaccination*	
None, n (%)	605 (85%)
Yes, n (%)	103 (15%)
Monoclonal antibodies’ usage (MABs), n (%)	40 (5.7%)
Days of hospitalization, median (IQR)	12 (8, 19)
Patients with > 30 days of hospitalization, n (%)	66(9%)
Patients treated with high-flow oxygen (HFO) supplementation, n (%)	295 (42%)
Patients admitted to intensive care unit (ICU), n(%)	96 (14%)
Death overall mortality, n (%)	36 (5.1%)
30-day mortality, n (%)	17(2.4%)

The median length of stay was 12 days (IQR: 8–19). Overall, in-hospital mortality was 5.1% (*n* = 36), while 30-days mortality rate was 2.4% (*n* = 17).

According to SARS-CoV-2 vaccination, 103/708 (14.6%) patients completed the vaccination schedule (Group B), while 605/708 (85.4%) patients (Group A) were not vaccinated or did not complete their schedule.

In Table 2, we reported the patients′ characteristics stratified by vaccination status. Patients of Group A and Group B significantly differ for age (66 years *vs*. 79 years, *p* < 0.01), prevalence of comorbidities (which were most frequent in Group B), need for HFO, which occurred more frequently in Group A (268/605, 44% *vs.* 27/103, 26%, < 0.01), and for the proportion of subjects who received treatment with MABs before being hospitalized (23/605, 3.8% *vs.*17/103, 17%, *p* < 0.01). It is also interesting to note that the median days from sympton onset to treatment significantly differ between the unvaccinated and vaccinated patients (7 days [IQR: 5-10] vs. 4 days [IQR: 3-7]; p<0.01), with patients of group B treated earlier than the unvaccinated. Moreover, ICU admission was significantly lower in vaccinated patients compared with those without vaccination (15% *vs.* 5%; *p* = 0.005).

**Table 2:** Patients’ characteristics at baseline according to vaccination status

	Vaccination status		*p* value^a^
	None, *N* = 605	Yes, *N* = 103	
Age, median (IQR)	66 (55, 77)	79 (66, 87)	** < 0.001**
Gender			0.074
Female, *n* (%)	198 (33%)	43 (42%)	
Male, *n* (%)	407 (67%)	60 (58%)	
Country			0.3
Italian, *n* (%)	528 (87%)	94 (91%)	
Foreign country, *n* (%)	77 (13%)	9 (8.7%)	
Days from symptoms to remdesivir, median (IQR)	7.0 (5.0, 10.0)	4.0 (3.0, 7.0)	** < 0.001**
Days from symptoms to remdesivir (categorical)			** < 0.001**
< = 5, *n* (%)	189 (31%)	67 (65%)	
> 7, *n* (%)	220 (36%)	11 (11%)	
6–7, *n* (%)	196 (32%)	25 (24%)	
Comorbidities, at least 1, *n* (%)	463 (77%)	93 (90%)	**0.002**
Comorbidities, three or more, *n* (%)	158 (26%)	63 (61%)	** < 0.001**
Hypertension, *n* (%)	292 (48%)	65 (63%)	**0.005**
Diabetes, *n* (%)	112 (19%)	30 (29%)	**0.013**
Obesity, *n* (%)	75 (12%)	9 (8.7%)	0.3
Neoplasm, *n* (%)	81 (13%)	21 (20%)	0.061
Cardiac disease, *n* (%)	206 (34%)	69 (67%)	** < 0.001**
Chronic respiratory diseases, *n* (%)	41 (6.8%)	16 (15%)	** < 0.001**
Other comborbidities, *n* (%)	173 (29%)	72 (70%)	** < 0.001**
MABs, *n* (%)	23 (3.8%)	17 (17%)	** < 0.001**
Days of hospitalization, median (IQR)	12 (8, 19)	12 (8, 20)	0.7
Need for HFO supplementation, *n* (%)	268 (44%)	27 (26%)	** < 0.001**
Need for transfer in ICU, *n* (%)	91 (15%)	5 (4.9%)	**0.005**
Death, *n* (%)	29 (4.8%)	7 (6.8%)	0.4

IQR: interquartile range; HFO: high-flow oxygen supplement; ICU: intensive care unit; n: number; %: percentage; MABs: monoclonal antibodies; ^a^Wilcoxon rank-sum test; Pearson's Chi-squared test. Statistically significant *p* values are shown in bold type

We did not find any statistically significant difference between the two groups in terms of mortality, gender, and ethnicity.

As previously mentioned, we performed a propensity score matching with logistic function to assess the impact of being vaccinated on the already mentioned outcomes. As a result, 196 patients out of 708 were retained and matched in two well-balanced groups of 98 patients each, while 512 patients were excluded from the analysis. The results yielded by the propensity score analysis (see Table 3) were also in favor of the vaccinated patients, confirming a statistically significant difference in terms of ICU admissions (Odds Ratio [OR] 0.27, 95%CI 0.08-0.75; p=0.02) and need for HFO supplementation (OR 0.42, 95%CI 0.22-0.79; p<0.01). Furthermore, being vaccinated seems to have a beneficial effect on the length of stay, which was significantly shorter (*p* < 0.01).

**Table 3:** Logistic regression model showing the association between covariates and three categorical variables: death, need for ICU, and need for high-flow oxygen supplementation after propensity score matching

	Death			ICU			HFO		
	OR	95%CI	*p* value	OR	95%CI	*p* value	OR	95%CI	*p* value
Vaccine	0.73	0.22, 2.38	0.6	0.27	0.08, 0.75	**0.018**	0.42	0.22, 0.79	**0.008**
Gender (Male)	0.89	0.25, 3.23	0.9	0.46	0.15, 1.33	0.2	1.51	0.77, 3.04	0.2
Age	1.09	1.02, 1.17	**0.016**	0.96	0.93, 1.00	0.055	1.00	0.98, 1.03	0.8
IPA	0.61	0.17, 2.28	0.4	1.02	0.33, 3.29	> 0.9	1.23	0.61, 2.51	0.6
Cardiac disease	2.58	0.57, 18.7	0.3	2.43	0.79, 8.68	0.14	1.41	0.68, 2.98	0.4
Diabetes	0.95	0.24, 3.21	> 0.9	1.67	0.53, 5.02	0.4	1.52	0.75, 3.06	0.2
Obesity	1.32	0.07, 8.90	0.8	1.28	0.17, 5.92	0.8	1.08	0.30, 3.46	0.9
Neoplasm	1.24	0.29, 4.45	0.8	0.55	0.08, 2.28	0.5	0.58	0.23, 1.38	0.2
Days to remdesivir	1.02	0.85, 1.17	0.8	0.88	0.72, 1.04	0.2	0.92	0.82, 1.01	0.11
MABs	2.95	0.66, 11.8	0.13	1.49	0.31, 5.62	0.6	1.16	0.45, 2.90	0.8

OR: odds ratio; CI: confidence interval; ICU: intensive care unit; HFO: high-flow oxygen supplement; IPA: arterial hypertension; MABs: monoclonal antibodies; days to remdesivir: days from onset of symptoms to remdesivir administration. Statistically significant *p* values are shown in bold type

Finally, in terms of toxicities, three adverse reactions were registered, determining the discontinuation of remdesivir: two patients developed a skin rash and one patient an acute hepatitis. In all these patients, no severe outcomes were reported, and their adverse reaction gradually healed.

## Discussion

Although more than 2 years have passed since the beginning of COVID-19 pandemic, an effective and specific anti-SARS-CoV-2 drug is still missing. To control viral replication, various strategies have been implemented, including the repurposing of drugs used to treat other infections with supposed antiviral properties. It is the case of remdesivir, originally developed for the treatment of Ebola and Marburg virus infections, which was subsequently investigated in randomized clinical trials, obtaining both the FDA and European Medicines Agency (EMA) “emergency” authorization for the treatment of COVID-19 in May and October 2020, respectively. In contrast, the WHO recently recommend against the use of remdesivir for hospitalized patients.

Despite the conflicting recommendations of the effectiveness of this drug for the treatment of COVID-19 pneumonia, a large-scale use of the drug emerged in clinical practice. Our real-life study enrolled patients with moderate-to-severe COVID-19 pneumonia and treated with remdesivir from September 2020 to January 2022, including both vaccinated and not-vaccinated individuals. To our knowledge, this is the first study that focuses on the possible relationship between remdesivir and SARS-CoV-2 vaccination on the outcomes of a cohort of COVID-19 patients. In our study, the overall in-hospital mortality was 5.1%, significantly lower both than that reported in the ACTT-1 study and other observational studies [25]. These findings suggested that in our study cohort, early treatment with remdesivir within 7 days from symptom onset has improved clinical conditions of patients with COVID-19 pneumonia and prevented their progression to a more severe respiratory disease. Indeed, preclinical study of remdesivir in animal models of SARS-CoV-2 infection demonstrated that early initiation of remdesivir significantly reduced viral loads in lung tissue [26], increasing its efficacy against the acute infections [27].

There was no significant difference in mortality between vaccinated and non-vaccinated patients, even if, in our cohort, vaccinated patients were significantly older and with a higher number of pre-existing comorbidities, two main determinants of COVID-19-related in-hospital mortality [28].

Our data are also consistent with recent reports in which mortality in vaccinated patients admitted to ICU was paradoxically even higher than in non-vaccinated patients precisely, but mainly due to the older age and the higher prevalence of comorbidities, especially cardiovascular ones [29, 30]. It should be noted that the mortality rate between the two groups did not change after the propensity score matching to reduce the effects of observed confounders [27].

In this study, the median time from vaccination to hospital admission was not known. However, the patient group who underwent a full vaccination course experienced a lower need for ICU admission (5% vs. 21%, *p* < 0.001) and for high-flow oxygen therapy (26% *vs.* 42%, *p* = 0.016). A recent published systematic review and meta-analysis reported that remdesivir was associated with a lower likelihood of requiring high-flow supplemental oxygen and invasive mechanical ventilation, compared to the placebo or standard care, but no data were available on the vaccination status of the patients [13].

Interestingly, in our cohort study, the interval between symptom onset and remdesivir administration in vaccinated patients was shorter (*p* < 0.01) than in non-vaccinated patients, confirming that timing of remdesivir treatment may be crucial for patient's clinical outcomes.

Overall, these data may suggest that, in the high-risk group of patients, the combination of monoclonal antibodies, with an early remdesivir treatment, and two-dose vaccination significantly prevents severe clinical progress of COVID-19 pneumonia and represents an effective therapeutic approach.

All our patients received remdesivir for a 5-day course according to Spinner et al*.* who demonstrated that patients who received remdesivir for 10 days had no additional benefit than those receiving a 5-day course (*p* = 0.18) [14].

Moreover, in accordance with the literature, remdesivir represented a safe therapeutic choice in our study, since only 3/708 (0.4%) patients experienced side effects [10].

Despite a recently published narrative review, however, summarized the safety and efficacy profiles of the various drugs used, as well as of the different vaccines, no data are available regarding the possible differences in response to remdesivir between vaccinated and unvaccinated patients, and no studies have investigated the possible correlation between vaccination, use of remdesivir and COVID-related outcomes [31].

An observational study on real-world data published in early 2023, on the other hand, investigated the differences, in terms of effectiveness measured through a composite primary endpoint of "in-hospital deterioration," between vaccinated and unvaccinated patients treated with remdesivir or sotrovimab [32]. Differently from our study, the authors dealt mainly with COVID-19 patients with mild-to-moderate symptoms rather than patients with an advanced state of disease. Evidence from this study suggests that remdesivir and sotrovimab may be beneficial when administered to unvaccinated patients in the early stages of moderate to prevent progression and no protective effect was found in vaccinated patients.

Our study has some limitations: the retrospective design and the restriction of remdesivir treatment to patients meeting Italian Health Ministry criteria for its use, limiting the extrapolation of results to other types of patients with COVID-19. In addition, it is a mono-center cohort study involving a wide but limited number of patients.

Lastly, a further limitation of the study concerns the role of VOCs. In our research, half of the patients were infected with the "Wuhan Strain" variant of the virus, whereas the remaining with the Alpha, Delta, and Omicron variants. Further stratification among different sub-variants and a contextual enlargement of the cohort could have helped to understand whether VOCs might have, in some way, influenced the clinical outcomes.

However, this is a study including individuals approached with a standardized treatment protocol and conducted over an extended observation period including the widespread use of vaccines and the emergence of variants such as Delta and Omicron, which could have had an impact on the patient’s clinical outcome. It is reasonable to question whether the use of antiviral therapy is warranted in these patients. Of note, an intriguing issue may be the possible role of gene mutations in this setting. Several recently published studies have suggested the possible involvement of structural modifications of epithelial cells [33–35]. This new evidence paves the way toward a new approach, potentially of the target therapy type, also in this clinical setting.

Finally, as far as we are concerned, we believe that observational studies collecting real-world data should be promoted, to accurately monitor the effectiveness and safety of new drugs in a rapidly and constantly evolving context such as a pandemic.

## Conclusions

Although raw mortality rate was similar between vaccinated and unvaccinated groups before the propensity score matching and remained similar even after matching the two groups for all baseline characteristics, our findings suggest that a complete vaccination course could have an impact in reducing the need for transfer in ICU and for HFO supplementation in patients with COVID-19 pneumonia treated with remdesivir. Further well-design clinical studies are recommended to confirm our results and new genomic approaches urge to be implemented to face possible future SARS-CoV-2 pandemic waves.

## Data Availability

Anonymized data are available upon request to the corresponding author.
